# Profunda Brachii Vein Revisited: Anatomy of the Vein in the Posterior Upper Arm and Its Relationship to the Triangular Interval

**DOI:** 10.7759/cureus.110997

**Published:** 2026-06-16

**Authors:** Hironobu Uzawa, Hiroto Kobayashi, Wataru Hashizume, Tsubasa Yamaguchi, Kyutaro Kawagishi

**Affiliations:** 1 Department of Anatomy and Structural Science, School of Medicine, Yamagata University, Yamagata, JPN

**Keywords:** anatomical variation, cadaver dissection, deep brachial vein, medical education, surgical anatomy, upper arm

## Abstract

Introduction: The profunda brachii vein (PBV) accompanies the profunda brachii artery through the radial groove and triangular interval. However, anatomical descriptions of the PBV remain limited and are sometimes inconsistent across textbooks and the literature, potentially leading to misunderstandings in medical education and surgical practice. Therefore, this study aimed to clarify the detailed anatomy of the PBV and classify variations in its course within the radial groove and triangular interval.

Methods: A total of 129 upper limbs from 65 adult Japanese cadavers (26 males and 39 females; mean age, 86.2 ± 7.9 years) were examined. The triceps brachii muscle was dissected and incised to expose the PBV traveling alongside the profunda brachii artery. The PBV was identified and classified according to its number, branching patterns, and drainage pathways. The diameter of the PBV was also measured. Variations in PBV classification and diameter were statistically analyzed according to side and sex.

Results: The PBV was present in all specimens and categorized into six types: (a) paired PBVs with a branch to the posterior circumflex humeral vein (PCHV) (33.3%); (b) a single PBV with a branch to the PCHV (27.9%); (c) a single PBV without a PCHV branch (15.5%); (d) paired PBVs without a PCHV branch (14.7%); (e) a single PBV terminating in veins other than the brachial or axillary veins beyond the triangular interval (5.4%); and (f) a single PBV draining directly into the PCHV without passing through the triangular interval (3.1%). Tributaries from the triceps brachii originated from the lateral head (93.0%), medial head (89.9%), and long head (50.4%). The median PBV diameter was 1.89 (interquartile range, 1.59-2.49) mm, with a maximum diameter of 6.35 mm and a minimum diameter of 0.68 mm. A significant side-to-side difference was observed (right: 1.93 (1.65-2.78) mm; left: 1.87 (1.49-2.45) mm; p = 0.044).

Conclusion: This study clarified the anatomy and its variations of the PBV, contributing to a better understanding of this vein.

## Introduction

**Figure 1:**
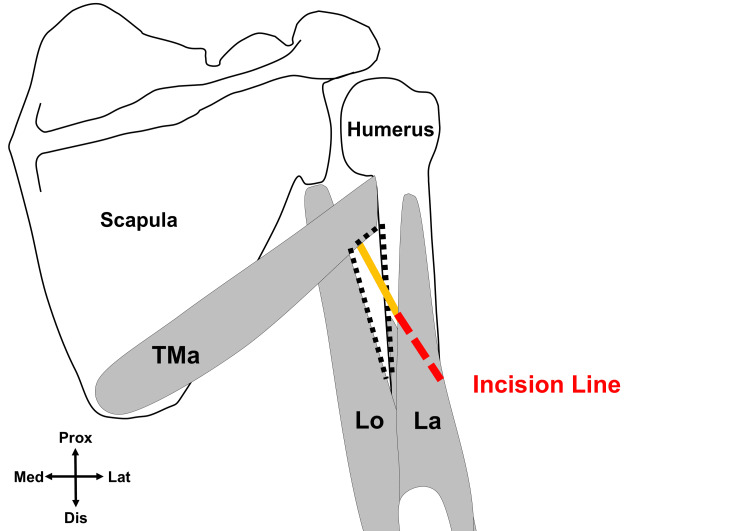
Dissection procedure to expose the profunda brachii vein (PBV). The black dotted triangle marks the triangular interval. The triceps brachii muscle was exposed, and the lateral head (La) was incised along the course of the radial nerve (yellow line) and the radial groove (red dashed line, incision line) to reveal the PBV and surrounding structures. Dis: distal side; Lat: lateral side; Lo: long head of the triceps brachii; Med: medial side; Prox: proximal side; TMa: teres major muscle; PBV: profunda brachii vein.

The profunda brachii vein (PBV) collects blood from the posterior upper arm and drains to the heart via the brachial and/or axillary veins [1,2]. However, the PBV has not been explicitly defined in the literature and is generally described as a vena comitans of the profunda brachii artery [2]. The profunda brachii artery originates from the brachial artery, passes through the triangular interval, courses along the radial groove with the radial nerve, and bifurcates near the lateral epicondyle into the middle and radial collateral arteries [3]. The triangular interval, also known as the lower triangular space, lateral triangular space, or triceps hiatus, is an important landmark for identifying the radial nerve and profunda brachii artery. It is bounded superiorly by the teres major, medially by the long head of the triceps brachii, and laterally by the humeral shaft (Figure 1) [4]. The PBV is assumed to follow the same route as the profunda brachii artery, with blood flowing in the reverse direction (distal to proximal), and drains into the brachial or axillary vein. It has also been reported to drain into variants of the axillary vein, including the accessory axillary vein [5-7].

However, previous anatomical sources have provided only vague or inconsistent descriptions of the PBV. Comprehensive anatomical textbooks include only implicit illustrations without detailed descriptions [2,8]. Textbooks for medical students omit the PBV entirely from both text and diagrams when discussing the triangular interval [9]. Likewise, a widely used anatomical atlas depicts the PBV without labeling it [4]. Older publications may also have introduced confusing terminology. Although the PBV is sometimes translated as the deep brachial vein, some articles have mislabeled the brachial vein itself as the “deep brachial vein,” likely because it is classified as a deep rather than superficial vein [10-13]. This may have contributed to the inconsistent use of the terms “deep brachial vein” and “profunda brachii vein.” Such confusion and limited anatomical descriptions may lead medical students to incorrectly assume that the PBV does not exist, resulting in misunderstandings of its anatomy and that of the triangular interval.

The PBV is also clinically relevant in muscle transfer and skin flap procedures [14-16], in which accompanying veins are harvested and anastomosed to maintain flap viability. Therefore, detailed knowledge of the PBV, including its anatomical variations in course and number, may aid surgical planning and facilitate the identification and preservation of collateral venous pathways during reconstructive and vascular procedures. Furthermore, a previous case report described the use of the PBV as a vascular access site for long-term hemodialysis patients when conventional venous access was unavailable [17]. Knowledge of the PBV diameter may be useful when assessing its suitability for vascular access, particularly in light of potential sex- and side-related differences.

Therefore, this study investigated the detailed anatomy of the PBV through cadaveric dissection, including its morphological characteristics, drainage patterns, and diameter. We also evaluated potential sex- and side-related differences. A clearer understanding of the PBV may improve anatomical education and support surgical planning.

## Materials and methods

Ethical considerations and cadaveric material

This cadaveric study was approved by the Research Ethics Committee of the Faculty of Medicine, Yamagata University (Approval No. 2024-157), and was conducted between April 2025 and May 2026. All cadavers were donated through living wills for medical education and research, with explicit consent for medical research participation. Inclusion criteria required written informed consent for postmortem research and family approval, while exclusion criteria were a history of surgery or significant structural damage to the posterior upper arm. The authors confirm compliance with local and international ethical guidelines and laws regarding the use of human cadaveric donors in anatomical research. Donor anonymity was maintained throughout the study.

A total of 130 upper limbs from 65 adult Japanese cadavers (26 males and 39 females; mean age, 86.2 ± 7.9 years) were examined. One left upper limb was excluded because of structural disruption from postmortem vascular extravasation, leaving 129 upper limbs (65 right and 64 left) for analysis.

Cadavers were embalmed with 10% formaldehyde injected through the left femoral artery. Skin and subcutaneous tissue from the scapular region to the posterior upper arm were removed, followed by dissection of the triceps brachii. The lateral head was incised along the radial nerve and radial groove to expose the PBV and surrounding structures. All dissections were performed carefully to preserve anatomical integrity (Figure 1).

Observations and analysis

In this study, the PBV was defined as a vein passing through the radial groove alongside the profunda brachii artery. Its course proximal to the radial groove was observed and classified. The frequency of each pattern was calculated as a percentage. Sex- and side-related differences in the distribution of PBV patterns were examined using the chi-square test. Furthermore, PBV tributaries were examined, and their occurrence was recorded.

The diameter of the PBV at its crossing point with the humerus was measured three times using a digital caliper (AD-5765A-150, A&D Company, Tokyo, Japan) by a trained assessor, and the mean of the three measurements was used as the representative value. Normality of the data distribution was assessed using the Shapiro-Wilk test. As the data were not normally distributed, side-to-side differences and differences between single and paired PBVs were analyzed using the Wilcoxon matched-pairs signed-rank test, whereas sex-related differences were analyzed using the Mann-Whitney U test. All statistical analyses were performed using GraphPad Prism version 9.5.1 (GraphPad Software, San Diego, California). Statistical significance was set at p < 0.05.

## Results

**Figure 2:**
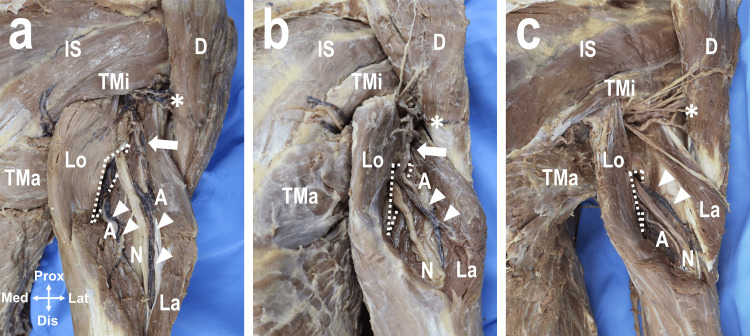
Representative cadaveric dissections of the PBV. The white dotted triangle indicates the triangular interval. The profunda brachii artery and radial nerve are labeled as “A” and “N,” respectively. The asterisk and white arrow indicate the posterior circumflex humeral vein (PCHV) and PBV’s collateral vein to the PCHV, respectively. Dis: distal side; IS: infraspinous; D: deltoid muscle; La: lateral head of the triceps brachii; Lat: lateral side; Lo: long head of the triceps brachii; Med: medial side; Prox: proximal side; TMa: teres major; TMi: teres minor; PBV: profunda brachii vein.

**Figure 3:**
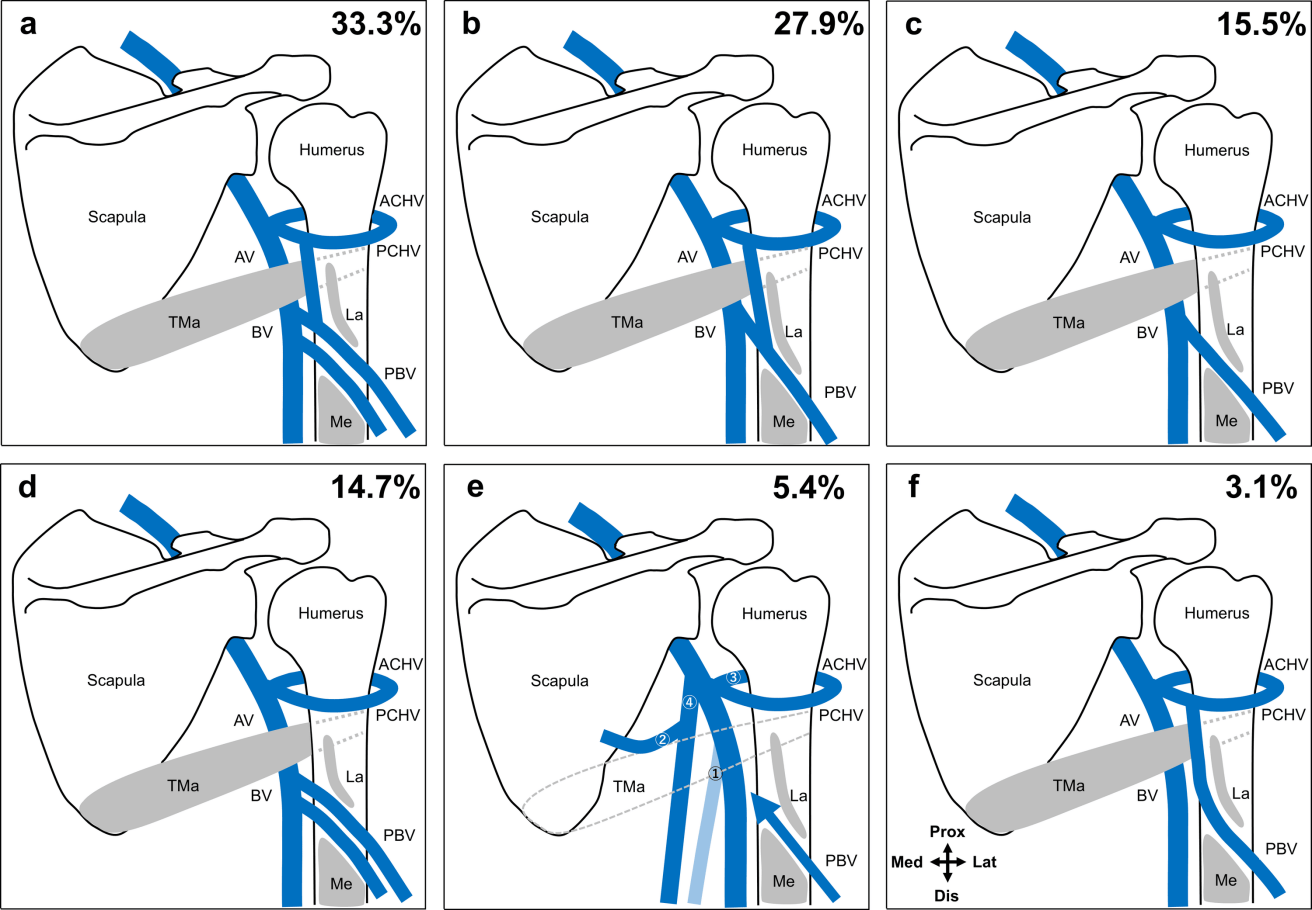
Classification of the PBV. Classification of the PBV into six types (a-f). Details of the classifications and the circled drainage sites are provided in the Results section. Blue lines represent deep veins, the light blue line represents a superficial vein, and gray areas represent muscle attachment sites. ACHV: anterior circumflex humeral vein; AV: axillary vein; BV: brachial vein; Dis: distal side; Lat: lateral side; Med: medial side; PCHV: posterior circumflex humeral vein; Prox: proximal side; TMa: teres major muscle; PBV: profunda brachii vein.

Representative examples of the PBV are shown in Figure 2. The vein was confirmed in the radial groove alongside the profunda brachii artery in all 129 specimens, indicating a prevalence of 100%. PBVs were observed as single or paired vessels along the profunda brachii artery. Of 129 sides, 125 (96.9%) passed through the triangular interval. The PBV was classified into six types (Figures 3a-3f) based on the number and branching patterns.

In most cases (43 sides, 33.3%; Figures 2a, 3a), a paired PBV with a branch to the posterior circumflex humeral vein (PCHV) was identified. The second most common pattern (36 sides, 27.9%; Figures 2b, 3b) followed the same course as Figure 3a but consisted of a single PBV. The third most common pattern (20 sides, 15.5%; Figures 2c, 3c) showed a single PBV without branches. The fourth pattern (19 sides, 14.7%; Figure 3d) matched Figure 3c but with paired PBVs.

**Figure 4:**
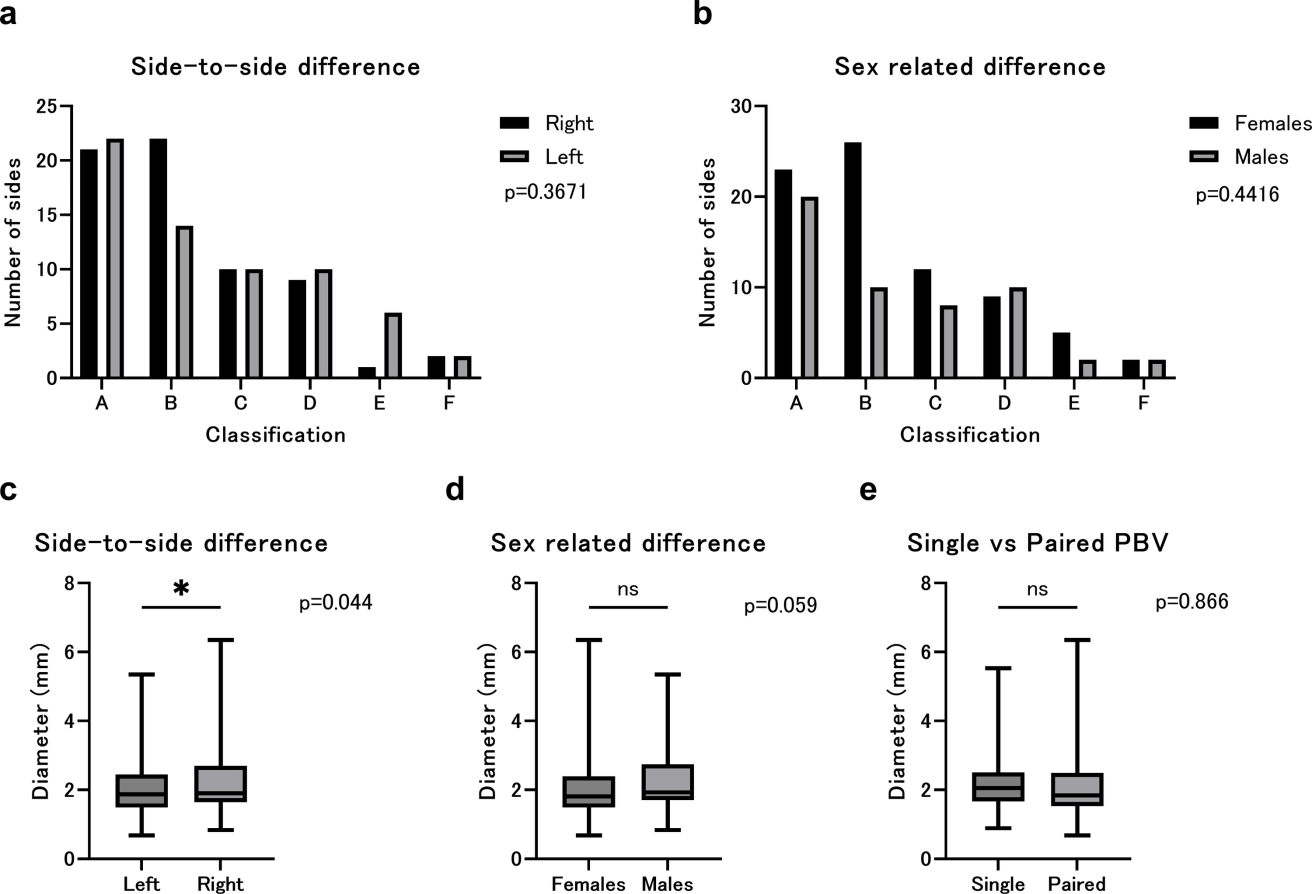
Statistical comparisons of PBV classification patterns and diameter. (a) Side-to-side and (b) sex-related differences in the classification patterns of the PBV. Comparisons of PBV diameter according to (c) side, (d) sex, and (e) single versus paired PBVs. *p < 0.05. PBV: profunda brachii vein.

In all cases shown in Figure 3a-3d, the PBVs terminated in the axillary or brachial vein. Rare variations in termination were observed in seven sides (5.4%; Figure 3e). The circled numbers indicate the PBV drainage sites: 1, basilic vein (three sides, 2.3%); 2, circumflex scapular vein (two sides, 1.6%); 3, anterior circumflex humeral vein (one side, 0.8%); and 4, subscapular vein (one side, 0.8%). Although the PBV passed through the triangular interval in 125 sides (96.9%; Figures 3a-3e), four sides (3.1%; Figure 3f) drained directly into the PCHV without passing through the triangular interval. No significant side-to-side or sex-related differences were observed in the distribution of PBV patterns (p = 0.3671 and p = 0.4416, respectively; Figures 4a, 4b).

The median PBV diameter was 1.89 (interquartile range, 1.59-2.49) mm. The maximum and minimum diameters were 6.35 mm and 0.68 mm, respectively (Table 1). A significant side-to-side difference was observed (right: 1.93 (1.65-2.78) mm; left: 1.87 (1.49-2.45) mm; p = 0.044; Figure 4c). In contrast, no significant sex-related difference (females: 1.81 (1.49-2.40) mm; males: 1.93 (1.70-2.74) mm; p = 0.059) or difference between single and paired PBVs (single: 2.06 (1.67-2.51) mm; paired: 1.92 (1.63-2.78) mm; p = 0.866) was observed (Figures 4d, 4e).

Proximal to the radial groove, the PBV received tributaries from the triceps brachii muscle with varying frequencies among its heads: the lateral head in 120 sides (93.0%), the medial head in 116 sides (89.9%), and the long head in 65 sides (50.4%). Only one side (0.8%) received a tributary from the short head of the biceps brachii, which belongs to the anterior compartment of the upper arm.

**Table 1:** Diameters of the PBV. Values are presented in millimeters (mm) as median (interquartile range) and range (minimum-maximum). PBV: profunda brachii vein.

	Median	Maximum	Minimum
Total	1.89 (1.59, 2.49)	6.35	0.68
Sides			
Right	1.93 (1.65, 2.78)	6.35	0.84
Left	1.87 (1.49, 2.45)	5.35	0.68
Sex			
Females	1.81 (1.49, 2.40)	6.35	0.68
Males	1.93 (1.70, 2.74)	5.35	0.84
Single versus Paired			
Single	2.06 (1.67, 2.51)	5.53	0.89
Paired	1.92 (1.63, 2.78)	6.35	0.68

## Discussion

This study aimed to investigate the anatomy of the PBV through cadaveric dissection. Detailed descriptions of the PBV are often omitted from anatomical textbooks, which may lead to confusion regarding its anatomy. For example, one anatomy textbook [9] neither illustrates nor mentions the PBV when describing the triangular interval. As a result, medical students potentially overlook the existence of the PBV. However, the present study identified the PBV in all specimens. The limited attention given to the PBV in anatomical texts may reflect its status as a vena comitans. Early anatomical texts [18] described veins as “following the course of the arteries, forming their venae comitantes,” and subsequent editions have provided similar descriptions. This historical perspective suggests that such veins were often omitted from illustrations and detailed descriptions to simplify anatomical presentation.

An anatomical textbook depicts the PBV as having two tributaries at the triangular interval [2]. Our data were partially consistent with this depiction, as the patterns shown in Figures 3a, 3d were identified in 62 sides (48.1%). However, a single PBV, as shown in Figures 3b, 3c, was identified in 56 sides (43.4%). This discrepancy may be explained by the greater anatomical variability of veins. Although not specifically related to the PBV, studies of the jugular vein [19] and upper-limb vasculature [20] have reported substantial venous variations. Such variability in venous anatomy may be attributable to venous malformations occurring during the late stages of embryonic development [21].

We identified several anatomical similarities between the PBV and the profunda brachii artery. Previous studies reported that 92.9% of profunda brachii arteries originate from the brachial or axillary artery [20]. Correspondingly, most PBVs drained into the brachial or axillary veins (118 sides, 91.5%; Figures 3a-3d). The PBV primarily received tributaries from the triceps brachii muscle, including the lateral head in 120 sides (93.0%), the medial head in 116 sides (89.9%), and the long head in 65 sides (50.4%). A previous narrative review [3] reported that the profunda brachii artery also gives off branches to the triceps brachii muscle, although frequencies were not quantified. These findings suggest that the PBV closely parallels the anatomical distribution of the profunda brachii artery. In four sides (3.1%), the PBV drained into the PCHV (Figure 3f). Similarly, previous studies reported that the profunda brachii artery originates from the posterior circumflex humeral artery in 8.4% of cases [22,23]. Collectively, these findings support the anatomical relationship between the PBV and the profunda brachii artery.

Regarding vessel diameter, our results showed that the PBV had a median diameter of 1.89 (1.59-2.49) mm, and significant side differences were identified. According to vascular access guidelines, a vein with a diameter of ≥2.5 mm is generally considered suitable for the creation of vascular access in patients undergoing hemodialysis [24]. In the present study, the median diameter of the PBV was smaller than this recommended threshold. However, the maximum diameter observed was 6.35 mm, and 48.1% of specimens (Figure 3a, 3d) exhibited paired PBVs, suggesting the presence of venous collateral drainage. Therefore, the PBV may be considered a potential alternative site for vascular access in selected patients, particularly long-term hemodialysis patients in whom conventional veins are unavailable [17], although side differences in vessel diameter should be taken into consideration.

Furthermore, knowledge of the anatomical course and variations of the PBV may be useful in muscle or skin flap procedures [14-16], as preservation of venous drainage is critical for flap viability. The present findings may assist surgeons in anticipating venous anatomy during flap elevation and transfer. In addition, our data showed that no significant differences in PBV course patterns were observed between sides or sexes, suggesting that these factors may not require special consideration during preoperative planning.

One limitation of this study is that all cadavers were from elderly individuals; therefore, PBV anatomical patterns may differ in younger populations. However, to our knowledge, this is the first study to comprehensively investigate PBV anatomy. Our findings may improve anatomical understanding of this vein among medical students and clinicians.

## Conclusions

The present study demonstrated that the PBV is consistently present and exhibits variations in its course, diameter, and drainage patterns. Recognition of these variations may improve understanding of upper-limb vascular anatomy and help resolve inconsistencies in previous anatomical descriptions, particularly for medical students learning about the triangular interval. Furthermore, knowledge of the anatomy and diameter of the PBV may aid surgical planning, including reconstructive procedures such as skin and muscle flap transfer, and support the assessment of the PBV as a potential vascular access site.
